# Psychological symptoms and associated psychosocial factors among fathers attending a tertiary children’s hospital in Nepal

**DOI:** 10.1371/journal.pgph.0006535

**Published:** 2026-05-21

**Authors:** Nishchal Devkota, Ashwini Poudel, Bishesh Bhatta

**Affiliations:** 1 CiST College, Kathmandu, Nepal; 2 Nobel College, Kathmandu, Nepal; 3 National Open College, Lalitpur, Nepal; Universiti Kuala Lumpur Royal College of Medicine Perak, MALAYSIA

## Abstract

Little has been done to focus on paternal mental health in Nepal despite evidence that fathers’ depression, anxiety, and stress can hurt family functioning and child development. This research measured the prevalence of depressive, anxiety, and stress symptoms among fathers visiting a tertiary children’s hospital in Kathmandu and analyzed the related psychosocial variables.A cross-sectional analytical study was conducted at Kanti Children’s Hospital, Kathmandu, among 312 biological fathers of children under two years. Data on psychosocial factors, including stressful life events, financial burden, work-life balance, family support, partner communication, masculinity norms, and child health conditions, were collected using interviewer-administered questionnaires. Psychological symptoms were assessed using the Depression, Anxiety, and Stress Scale-21 (DASS-21), and masculinity norms using the Macho Scale. Descriptive statistics and multivariate logistic regression were used to estimate adjusted odds ratios (AORs) with 95% confidence intervals. Most fathers reported no psychological symptoms: depression (86.9%), anxiety (93.3%), and stress (95.8%). Moderate to severe depressive symptoms, moderate anxiety, and mild to moderate stress were reported by 4.1%, 3.8%, and 4.2% of participants, respectively. Stressful life events during the past year were significantly associated with depression (AOR 4.16, 95% CI: 1.90–9.14), anxiety (AOR 4.66, 95% CI: 1.22–17.84), and stress (AOR 4.07, 95% CI: 1.19–13.95). Lack of family support independently predicted depression, anxiety, and stress. Anxiety was also associated with financial burden and high machismo attitudes.Although most fathers reported low psychological distress, stressful life events, limited family support, financial burden, and traditional masculine norms were significantly associated with depression, anxiety, and stress. Family-centered mental health services and routine screening in pediatric settings may help identify and support vulnerable fathers.

## Introduction

The mental health of fathers has long been in the shadow of the research on maternal mental health, although there is emerging evidence of high prevalence of depression symptoms, anxiety, and stress in men during the critical parenting period [1,2]. As per epidemiological studies, levels of psychological distress amongst fathers are high, and adverse impacts on child development and family functioning have been connected to anxiety and depression of fathers [3,4]. An extensive meta-analysis that correlated the indicators of parental anxiety and depression with deteriorated social, emotional, and cognitive outcomes in children showed that poor mental health of males in the familial context is exceptionally significant [5]. Along with a lack of social support, unemployment and perceived stress make one more vulnerable to mental health problems in the postnatal period, and financial difficulties and negative life experiences often reveal themselves as a risk factor of elevated depression symptoms in the parental population [6]. Besides, cross-sectional findings demonstrate the importance of socioeconomic context in the parental mental health risk patterns by revealing that depressive symptoms correlated with poor wealth status, loneliness, and perceived family income stress in fathers in community samples significantly [7]. On top of the economic factors, psychosocial factors are also of importance. The work-family conflict and ineffective work-life balance have also been associated with a high level of psychological uneasiness in the male parents due to cultural and societal expectations and role pressure during the years of child-rearing [8]. Moreover, the presence of stressors in depressive symptomatology of males may be enhanced by gender role-appropriate behavior, which may discourage seeking help and enhance the internalization of stress. Such norms encompass the classical traditional male values of emotional restraints and independence [9,10]. The narrow idea of machismo has not been operationalized as much in parental mental health studies as compared to its expression as machismo, but rigid gender norms have been associated with poorer mental health outcomes in men [9–11]. It has been established that psychological suffering in men is minimized by the presence of protective factors such as planned pregnancy and good family support [11,12]. Home base and social support may offer emotional hedges against the impacts of pressures and promote greater coping during life transitions, such as parenthood [5]. Although there is increasing evidence of paternal mental health challenges in the world, little has been done regarding research about fathers in Nepal. Most of the Nepalese studies have focused on maternal or overall adult mental health and not on fatherhood proactively. Yet, the fact is that depression and anxiety are widespread in Nepali people, and cause disability and poor quality of life [13–16]. Parental depression, along with nutritional outcomes, was found in a study of the Nepal household survey (cross-sectional study), and Nepalese fathers are less likely to get depressed compared to mothers, but it still could influence family life and the well-being of children [17].

Paternal mental health is a critical, yet overshadowed, determinant within Nepal’s health system since fathers often guide household health-seeking behavior and health outcomes for the family [17]. Moreover, in the perinatal period, a fathers’ psychological state is a key predictor of adverse pregnancy outcomes and maternal recovery [1,4]. Left untreated, paternal distress can exacerbate a cycle of intergenerational developmental delays [3,5]. Additionally, deep-rooted traditional masculinity among men in Nepal often leads to delayed clinical presentation and increased symptom severity [9,10]. Currently, the Nepalese health system lacks a formal, father-inclusive mental health screening protocol. While community-based studies have explored parental well-being in specific districts, these are often fragmented and have not been integrated into national policy [6,13,18]. Consequently, fathers represent a silent demographic in primary care, struggling with symptoms that are neither proactively identified by providers nor reported by the men themselves due to social stigma [8,11]. The present paper tests the depressive, anxiety, and stress symptom levels among fathers with the help of the DASS and compares them with their relationships to the psycho-social factors. By assessing the unique associates of paternal suffering, this study attempts to fill a local service gap by providing the evidence-based foundation necessary to move forward for ‘family-centered’ care models [1,12,18]. Information gained in this study by finding important associates of paternal psychological suffering will be used to educate early intervention tactics and family-focused mental health programs, facilitating the integration of paternal screening into the existing maternal health care frameworks.

## Methodology

### Study area and period

The study took place at the Kanti Children Hospital, which is the sole government tertiary-level referral center regarding pediatric care in Nepal, in Kathmandu, and data was gathered between July and August 2024. The hospital offers outpatient and inpatient care to patients throughout the country in a broad range of child illnesses, including severe and chronic illnesses. The fathers of the outpatient department were invited to participate, and it was possible to examine the psychological symptoms and psychosocial variables in caregivers. Conducting the study in this hospital allowed the capture of experiences of directly involved fathers regarding the care of children with various medical conditions, representing typical and complex caregiving situations in Nepal.

### Study design, population & eligibility

The study adopted cross-sectional analytical design, which is an observational study where the measurements of variables of exposures (stressful events in past 1-year, financial burden, work life balance, family support, communication with partner, masculinity norms, chronic child health condition, planned pregnancy and socio demographic characteristics) and outcomes (e.g., depression, anxiety, stress) were conducted at one time. This approach has allowed computing the prevalence and analyzing relationships between psychosocial factors and the presence of psychological symptoms; it is not applicable to infer causation. The eligibility criteria included that the participants should be biological fathers between the ages of 18 and 65 years with at least one child below the age of two years who could provide informed consent in Nepali. Participants were specifically required to be in paid employment to assess the impact of the work-life balance & financial burden, which are the key stressors in the Nepalese context [12,19]. Further, restricting the sample to the paid employment ensures a level of socio-economic homogeneity, reducing the confounding influence of the acute financial instability on dire mental health outcomes [20]. Adoptive and step-fathers, fathers with a severe mental illness under treatment, and those who could not speak Nepali were not included. In the course of the research, all the participants were selected in the Outpatient Department (OPD) of Kanti Children’s Hospital. The unit of analysis was individual fathers.

### Sample size and sampling technique

The calculation of sample size was done using the standard method of calculating a proportion in an infinite population with 95 percent confidence and five percent margin of error. According to the Arghakhanchi study, Nepal [18] stated that 24% of the parents had anxiety prevalence; 280 was the minimum sample size needed. The final target respondent population was 312 respondents, having considered 10% non-response rate. This hospital-based study employed a consecutive purposive selection technique in choosing the respondents who were fathers. Under this technique, all the eligible fathers who met the inclusion criteria during the data collection period were enrolled until the target sample size was achieved.

### Data collection and analysis

Face-to-face, interviewer-administered survey questionnaires were used to obtain primary data. The survey provided data on the sociodemographic and family background of the participants, traditionally measured the Masculinity norms through the Macho Scale assessment, and assessed the extent of the symptoms of depression, anxiety, and stress (DAS) through the Depression, Anxiety, and Stress Scale -21 items (DASS-21) scale. Other work-life balance questions, family support, last year’s stressful experiences, financial burden, and pregnancy planning status were self-constructed by researchers and coded as binary (yes/no) responses; interviewer-standardized probes and interest were used with each item to ensure clarity, completeness, and consistency of answers. To simplify the analysis, age was divided into a mean to use as a cutoff age. This approach was adopted to enhance clinical interpretability and to facilitate the calculation of the odds ratio (OR) for identifying distinct risk strata. While we acknowledge that dichotomization can reduce statistical precision, the mean was selected as an objective, sample-specific threshold to ensure balanced group sizes comparison. Similarly, communication with spouse and macho attitude were dichotomized into two. Testing of 10% of the sample in a similar health institution was done to determine how clear, feasible, and flowing the questionnaire was. In the previous research, DASS-21 has been extensively applied and tested in Nepal [19–23]. The Macho Scale has been borrowed [24] (University of California, San Diego) with the official permission to use in the Nepali context; the items have been examined on the basis of cultural orientation and fitting to the existing stereotypes of gender. The DASS-21 and Macho Scale were administered in English to participants to make the constructs and scoring procedures similar. In the case of those who were not fully comprehending or needed some clarification on some items, the interviewer clarified them and guided them in the right direction to understand. All multi-item scales were evaluated on the basis of internal consistency by applying Cronbach’s alpha and proved to be reliably sound. The measurement validity of the study was supported by the use of previously validated instruments, adaptation of the instruments to the context, and standardization of the data collection procedures. The information was entered into Microsoft Excel and analyzed using SPSS version 22 (S1 Data). Participants’ socio-demographic and family-related characteristics, masculinity norms, and the level of depression, anxiety, and stress were summarized with the help of descriptive statistics. DAS outcomes were dichotomized to represent the presence or absence of clinically significant symptoms. The latter was defined as any score falling within the mild, moderate, or severe categories (cut-offs: Depression ≥ 10, Anxiety ≥ 8, and Stress ≥ 15). Bivariate logistic regression was used to test associations between categorical exposure variables and binary mental health outcomes. Multivariate logistic regression analysis was conducted to estimate adjusted odds ratios of the variables of significance using confidence intervals of 95 percent.

### Ethical considerations

The paper was conducted ethically (Reg. No 080/81/360) under the supervision of an Institutional Review Committee at Nobel College, Kathmandu, Nepal. The hospital administration gave formal permission. Additional verbal and written informed consent were received from all participants. This was on a voluntary basis, and one had the right to withdraw. To ensure confidentiality, data were anonymized, safely stored, and aggregated to report, in accordance with the recommendations of observational cross-sectional research. Conduct of the study was made in line with the ethical standards and the reporting as advised by the STROBE checklist to make it clear and transparent (S1 File).

## Results

The mean age of the respondents was 31.98 years (SD = 5.11), where 178 (57.1) respondents were below, and 134 (42.9) were above the age of 32. These were Hindu 255 (81.7%), Buddhist 37, Christian 15, and Muslim 5. The participants were of Ethnicity Brahmin/Chettri 141 (45.2%), Janjati 118 (37.8%), Dalit 36 (11.5%), Madeshi 12 (3.8%), Muslim 4 (1.3), and other ethnic groups 1 (0.3). The educational attainment of the participants was as follows: 60 (19.2) possessed less than a high school education, 178 (57.1) possessed a high school education, 52 (16.7) possessed a Bachelor’s degree, and 22 (7.1) possessed a Master’s degree. The number of participants with one child was 175 (56.1%), with two children was 115 (36.9%), with three children was 15 (4.8%), and with four children was 7 (2.2%). In terms of spousal communication, the participants who reported very good communication were 114 (36.5), good communication were 157 (50.3), moderate communication were 40 (12.8), and poor communication were 1 (0.3). (Table 1)

**Table 1:** Sociodemographic and Family Characteristics of Respondents (n = 312).

Variable	Category	n (%)
**Age**	Up to 32 years	178 (57.1)
	More than 32 years	134 (42.9)
**Religion**	Hindu	255 (81.7)
	Buddhist	37 (11.9)
	Christian	15 (4.8)
	Muslim	5 (1.6)
**Ethnicity**	Brahmin/Chettri	141 (45.2)
	Dalit	36 (11.5)
	Janjati	118 (37.8)
	Madeshi	12 (3.8)
	Muslim	4 (1.3)
	Others	1 (0.3)
**Educational Level**	Less than high school	60 (19.2)
	High school	178 (57.1)
	Bachelor’s	52 (16.7)
	Master’s	22 (7.1)
**Number of Children**	1	175 (56.1)
	2	115 (36.9)
	3	15 (4.8)
	4	7 (2.2)
**Spousal Communication**	Very good	114 (36.5)
	Good	157 (50.3)
	Moderate	40 (12.8)
	Poor	1 (0.3)

Out of 312 respondents, 271 (86.9), 28 (9.0), 12 (3.8), and 1 (0.3) had normal, mild to moderate, moderate, and severe levels of depression, respectively. In anxiety, the participants in the normal range were 291 (93.3%), those with mild and moderate anxiety were 9 (2.9%), those with moderate anxiety were 12 (3.8), and those with severe anxiety were 0 (0.0%). In terms of stress, the percentage of participants in the normal category was 299 (95.8), the percentage of participants in the mild to moderate category was 13 (4.2), the percentage of participants in the moderate stress category was 0 (0.0), and the percentage of participants in the severe stress category was 0 (0.0) as well. Concerning the machismo attitude, the normal participants were at 209 (67.0%), medium (31.7%), and high (1.3), respectively. The participants who had experienced stressful events within the last year were 47 (15.1), and those who had not experienced stressful events were 265 (84.9). The respondents with financial burden in the family were 93 (29.8), and those without financial burden were 219 (70.2). There were 289 (92.6 Percent) planned pregnancies and 23 (7.4 Percent) unplanned pregnancies. The number of respondents who provided positive reports on children with chronic or critical health problems was 77 (24.7%), and the number of those who did not have such problems was 235 (75.3%). The percentage who reported the ability to balance work-life was 283 (90.7), and those who could not balance work-life were 29 (9.3). The number of respondents who had family support during problem solving was 275 (88.1%), and the number of respondents who had no family support was 37 (11.9%). (Table 2)

**Table 2:** DAS Distribution and Psychosocial Factors (n = 312).

Variable	Normal n (%)	Mild n (%)	Moderate n (%)	Severe n (%)	Yes n (%)	No n (%)
Depression	271 (86.9)	28 (9.0)	12 (3.8)	1 (0.3)	—	—
Anxiety	291 (93.3)	9 (2.9)	12 (3.8)	—	—	—
Stress	299 (95.8)	13 (4.2)	—	—	—	—
Macho category	209 (67.0)	99 (31.7) (medium)	—	4 (1.3) (high)	—	—
Experienced stressful events in the past 1 year	—	—	—	—	47 (15.1)	265 (84.9)
Financial burden in the family	—	—	—	—	93 (29.8)	219 (70.2)
Planned pregnancy	—	—	—	—	289 (92.6)	23 (7.4)
The child has a chronic/critical health issue.	—	—	—	—	77 (24.7)	235 (75.3)
Able to balance work–life situation	—	—	—	—	283 (90.7)	29 (9.3)
Family support in problem-solving	—	—	—	—	275 (88.1)	37 (11.9)

Binary logistic regression tests were also done to determine the factors at risk of depression, anxiety, and stress. Odds ratios were estimated, unadjusted and adjusted, with 95% confidence interval. All three outcomes were always related to experiencing stressful events in the past year. In the crude model, stressful events were associated with the increased probability of depression (UOR = 4.95, 95% CI: 2.38-10.28), anxiety (UOR = 3.97, 95% CI: 1.54-10.21), and stress (UOR = 5.39, 95% CI: 1.72-16.85). These associations were statistically significant even after accounting for the confounding factors: depression (AOR = 4.16, 95% CI: 1.90-9.14), anxiety (AOR = 4.66, 95% CI: 1.22-17.84), and stress (AOR = 4.07, 95% CI: 1.19-13.95). The absence of family support was also strongly and consistently related to all three conditions. Patients with no family support were more likely to be depressed (UOR = 4.77, 95% CI: 2.19-10.42), anxious (UOR = 5.56, 95% CI: 2.12-14.52), and stressed (UOR = 10.46, 95% CI:3.30-33.16). These were found to be important in the adjusted depression (AOR = 3.36, 95% CI: 1.42-7.94), anxiety (AOR = 4.02, 95% CI: 1.08-14.93), and stress (AOR = 7.22, 95% CI: 2.13-24.49) models. All three outcomes were significantly related to financial burden, with unadjusted results of depression (UOR = 2.29, 95% CI: 1.17-14.49), anxiety (UOR = 3.45, 95% CI: 1.40-8.51), and stress (UOR = 4.02, 95% CI: 1.28-12.66). Having adjusted, financial burden continued to show a significant relationship with anxiety only (AOR = 4.34, 95% CI: 1.22-15.46), whereas its relationship with depression (AOR = 2.04, 95% CI: 0.99-4.21) and stress (AOR = 3.00, 95% CI: 0.88-10.18) became non-significant. The failure to balance work and life was significantly associated with depression in the unadjusted model (UOR = 3.53, 95% CI: 1.48-8.41), but the significance was not found after adjustment (AOR = 1.84, 95% CI: 0.68-4.97). Regarding anxiety-specific factors, after the unadjusted analysis, moderate intensity of communication with a partner was linked to greater odds of anxiety (UOR = 3.77, 95% CI: 1.42-10.02), which was not the case in the adjusted model (AOR = 1.83, 95% CI: 0.47-7.12). Attitude of high machismo, however, was a great independent predictor of anxiety in both unadjusted (UOR = 4.18, 95% CI: 1.42-12.23) and adjusted analyses (AOR = 5.25, 95% CI: 1.47-18.75). Only in the unadjusted model, chronic child health issues were related to anxiety (UOR = 3.03, 95% CI: 1.23-7.46), but not after the adjustment (AOR = 1.86, 95% CI: 0.53- 6.44). (Table 3).

**Table 3:** Factors associated with depression, anxiety, and stress (n = 312).

Variable	Category	Depression		Anxiety		Stress	
		UOR (95% CI)	AOR (95% CI)	UOR (95% CI)	AOR (95% CI)	UOR (95% CI)	AOR (95% CI)
Stressful events (past 1 year)	Yes	4.95 (2.387 –10.28) *	4.16 (1.90 –9.14) *	3.97 (1.54 –10.21) *	4.66 (1.22 –17.84) *	5.39 (1.72 –16.85) *	4.07 (1.19 –13.95) *
	No	Ref.	Ref.	Ref.	Ref.	Ref.	Ref.
Financial burden	Yes	2.29 (1.17 –4.49) *	2.04 (0.99 –4.21)	3.45 (1.40 –8.51) *	4.34 (1.22 –15.46) *	4.02 (1.28 –12.66) *	3.00 (0.88 –10.18)
	No	Ref.	Ref.	Ref.	Ref.	Ref.	Ref.
Maintaining Work–life balance	Not able	3.53 (1.48 –8.41) *	1.84 (0.68–4.97)	—	—	—	—
	Able	Ref.	Ref.	—	—	—	—
Family support	No	4.77 (2.19 –10.42) *	3.36 (1.42 –7.94) *	5.56 (2.12 –14.52) *	4.02 (1.08–14.93) *	10.46 (3.30 –33.16) *	7.22 (2.13 –24.49) *
	Yes	Ref.	Ref.	Ref.	Ref.	Ref.	Ref.
Communication with the partner	Moderate	—	—	3.77 (1.42 –10.02) *	1.83 (0.47 –7.12)	—	—
	Good	—	—	Ref.	Ref.	—	—
Macho category	High	—	—	4.18 (1.42 –12.23) *	5.25 (1.47 –18.75) *	—	—
	Low	—	—	Ref.	Ref.	—	—
Chronic child health issues	Yes	—	—	3.03 (1.23 –7.46) *	1.86 (0.53 –6.44)	—	—
	No	—	—	Ref.	Ref.	—	—

UOR = Unadjusted odds ratio; AOR = Adjusted odds ratio; CI = Confidence interval; Ref. = Reference category; *Statistically significant if the 95% CI does not include 1; ‘—’ indicates the variable was not included in that outcome model.

## Discussion

The study revealed that most of the fathers whose children were receiving treatment in a tertiary children’s hospital in Nepal were psychologically doing well. Only a small percentage showed emotional distress: approximately 4% had moderate to severe depressive symptoms, the same percentage had moderate anxiety, and slightly more than 4% had to face mild stress. These are significantly lower than the reported figures of the general population in South Asian and Nepali research, with between a fifth and over half of individuals frequently presenting significant psychological problems [25–27]. The comparatively low distress level can be explained by the fact that the sample is mostly made up of healthy, working-age males; some of the fathers might have been understating the difficulties because of the cultural norms and values of masculinity, or they could respond more intensely and resiliently to those challenges due to the necessity to accompany their children to the hospital.

Despite nearly one-quarter of fathers reporting that their child had a chronic or critical illness, overall levels of paternal depression and anxiety were low. Persistent health problems in pediatrics were found to be correlated with greater paternal anxiety in unadjusted analysis; however, this association was not significant after adjusting for the variables that may have caused the association. This means that the relationship can be explained by other correlated factors. The general under-prevalence of the symptoms may be due to effective coping strategies or healthy family support; it can also be a sign of the under-awareness or under-reporting of the emotional problems. Previously, research in the Neonatal Intensive Care Unit [28] setting has continuously identified infant morbidity and emergency department hospitalization as highly important stressors to parents, especially the fathers, which is due to the parenting demands, the lack of knowledge, and economic burden. Similarly, research findings in urban Bangladesh [29] also reveal that parents with children who have chronic illnesses are more likely to have symptoms of depression. Combined, though our refined results do not suggest independent correlation, dads of children with severe health problems can still represent a psycho-vulnerable group, and their anguish may go unnoticed in aggregate studies.

In this study, lack of family support was associated with depression, anxiety, and stress among the strongest ones. Even after adjustment, perceived family support continued to have a direct significant impact on all three outcomes, which highlights its primary protective value as far as paternal mental health is concerned. Almost nine out of ten fathers said that their family assisted them in resolving their problems, and a comparable percentage of those said that they could manage family and work challenges. This is reflected in South Asia and Pakistan, where a lack of social support and marital stressors are always found to be significant factors leading to perinatal depression [30,31]. The studies in South Asia and other Western nations also reveal that social support cushions the parents against psychological distress [12,25]. In Nepal, parents whose close relatives provide their support to them have significantly less distress, even when they encounter significant stressors like child migration [25].

Weak communication with the partners, inability to balance between work and family life, and paternal emotional distress that has been noted in the present study are also upheld by the previous literature as relationship dissatisfaction and work-family conflict are known to be reliable predictors of mental distress in their fathers [6,32–34]. However, perceived work-life balance in the current study is only linked to reduced levels of depression in unadjusted analyses and is no longer significant after adjustment, implying that its ostensible influence might be mediated by family or socioeconomic factors in general.

The significance of favorable settings has been supported by the results of Beni Municipality, where over 60% reported psychological distress among teachers working in public schools and over half reported occupational stress, mostly caused by low salaries and large workloads [35]. The most common in that case was distress, which occurred mainly when job demands exceeded resources and support. Collectively, such findings substantiate the finding that powerful informal family support networks, as opposed to work-life balance, are truly protective factors of the mental health of fathers, especially in a setting like Nepal, where formal mental-health services are still inaccessible [36].

In this paper, about one-third of fathers did not strongly subscribe to traditional machismo attitudes, whereas about two-thirds of fathers were placed in the category of mild machismo, with a small subgroup (1.3) having strongly macho attitudes. These findings are supported by a substantial amount of international research, according to which the traditional masculine standards, including self-reliance, emotional control, and toughness, correlate with lower help-seeking and the increased propensity of men to report on depression and psychological distress symptoms underreporting [12]. South and East Asian cultures, where men are expected to be steady providers and emotionally strong, may also contribute to the devaluation of emotional problems even when men experience financial pressures, migration stress, or are involved with caretaking duties [37,38].

The low rates of depression and anxiety observed in the current research ought to be viewed in this respect. Given the face-to-face interviewer-administered format of the study, social desirability bias may have influenced the results, as fathers who are more affiliated with machismo norms might downplay or hide emotional distress instead of reporting it. This tension is pointed out through qualitative research among Nepali migrant fathers who reported experiencing guilt, worry, and stress over children and having high social pressure to appear successful and emotionally robust as providers [37]. Even though the migration of fathers was not evaluated in this study, similar masculinity norms and gendered anticipations can be applied to urban Nepali paternal fathers navigating a tertiary hospital environment. These cultural pressures, compounded by the presence of an interviewer, may create a discrepancy between the perceived and recorded distress.

Remarkably, the greater the support of machismo tendencies in the given research, the more anxiety would be elevated, which supports the worries about the mental costs of strict masculine standards. This result correlates with evidence in Australia, where masculine gender role stress is one of the strongest predictors of paternal perinatal distress [32], and qualitative research in Singapore, which discusses the way men often suppress emotional problems because social norms of manhood require them to be strong and competent providers [39]. Combined, these results align with masculine standards that not only increase anxiety but also generalize it, which underlines the necessity of father-inclusive mental health services sensitive to the gendered expectations.

In this study, stressful life events within the past year was the most consistent predictor of paternal mental health, and remained significantly associated with depression, anxiety, and stress after adjusting for possible confounders. This is similar to fathers in the context of the COVID-19 pandemic, where the recent stressful situations were also associated with a high level of depressive and anxiety symptoms [40]. Similar meta-analytic results indicate that perceived stress is a combined risk factor of paternal post-partum depression [41], and the research on expectant fathers in Thailand has reported that perceived stress was a major predictor of depressive symptoms [29]. These data suggest that recent stress exposure is a primary and significant contributor to paternal psychological distress, both across cultural backgrounds.

A finer trend was shown by financial burden. It was bivariately related to depression and perceived stress, but was significant only in multivariate models in relation to anxiety. Such a pattern could be the sign that financial strain is manifested at the beginning as general distress and low mood, but as the effects of other stressors are considered, it also leads to anxiety. Preceding systematic reviews indicate that financial instability and unemployment levels predispose paternal depression [41], and longitudinal data demonstrate that fathers who lack healthcare employment due to financial reasons have mental health outcomes marked by negative trajectories [30]. This is, though, interesting, although only approximately 30 percent of the fathers in the current study reported a mental state of financial strain; this is noteworthy, of course, considering that several studies have consistently shown that financial strain is strongly associated with psychological distress in South Asia. The Nepali parents abandoned by migrant children, such as those, are more distressing in the background of health-related issues and with poor support services, in which financial support by the children is a less significant issue [25]. Adolescents in families with no savings or low socioeconomic status are at a higher risk of experiencing anxiety and depression [26], and young survivors of cancer in Asia generally mention financial distress as one of their greatest psychosocial problems [42].

The consistency of work-life balance with depression in bivariate analysis was found, but not with anxiety or stress, and it lost significance after adjustment. This would imply that the observed role of work-family balance on the symptoms of depression can be attributed in large part to more immediate stressors like recent life events and financial strain. This observation is contrary to the results observed in Kathmandu Valley, where over three-quarters of working dads indicated that they experienced hardships in achieving work-life balance, attributing it to considerable stress levels, poor work environment, unrealistic expectations, and lack of job control [43]. The somewhat positive image in the present sample seems to be due to more economic stability, or extended family helping with childcare and financial burdens, or social pressures to appear successful in coping, which, of course, causes fathers to present themselves as doing well.

Family support was found to be a protective factor in all three outcomes and was correspondingly related to depression, anxiety, and perceived stress after adjustment. This reinforces the importance of family relationships in buffering paternal mental health. Conversely, the association of communication with one partner was only related to anxiety in the bivariate analysis, implying that communication-related problems might indeed increase worries and tension, but when other factors are accounted for, their influences might be confounded with the broader family processes.

The macho attitudes were found to be uniquely linked with anxiety and relevant in the multivariate analysis, which means that strict gender norms can put fathers at risk of anxiety even without any other forms of stress. This type of attitude could diminish the expression of emotions and seeking help, which increases internalized distress. Only when compared to chronic child health problems, anxiety was found to have a significant effect on paternal mental health, indicating that the effect of family stress, caregiving burden, or financial strain may be indirect.

The majority of pregnancies in the study were planned, and more than 92% of the fathers stated that their child was wanted and was expected. This is essential since unplanned pregnancy is always associated with increased chances of postnatal depression across the globe, but planned pregnancies tend to indicate superior preparation, increased communication between the two partners, and support systems that are more stable. The extremely high proportion of intended pregnancy, thus, indicates a rather privileged and purposeful segment of parents in relation to the rest of the population.

Simultaneously, almost a quarter of fathers reported that their child has a chronic or critical health condition, which again was related to anxiety, at least in the unadjusted analysis, but was not notable after the control of other variables. South Asian studies indicate that the chances of depression in mothers are very high in the case of child’s long-term disease or an accident, [29]. The attenuation of this effect in the multivariate model might be an indication of the varied paternal roles that fathers usually play in caregiving. This means that mothers are directly more engaged in day-to-day medical and emotional assistance, and fathers, in turn, are more likely to be engaged in financial support. The psychological effects of a child’s sickness on fathers may be mediated by financial tension, family tension, and role anticipations instead of manifesting as discrete anxiety symptoms. This finding is in line with information on the East Asian region that the combination of role strain and financial pressure, combined with the culturally defined parenting roles, influences caregiver well-being [44].

Nepalese studies indicate that depression, anxiety, and stress have pervaded the youthful population and adults during and after the COVID-19 period. As an example, a study of Nepali youth determined that approximately fifty percent of the respondents displayed symptoms of anxiety, approximately half displayed symptoms of depression, and over fifty percent exhibited high stress [27]. Almost one out of every five parents who had migrant children has also reported psychological distress [25], and more than 60% of the teachers in Beni Municipality were reported to be psychologically distressed [35]. Against this backdrop, fathers in the current study attending tertiary children’s hospital are much less distressed. On the whole, these data indicate that actively engaged fathers in the health care of their child, mostly unplanned pregnancies, good family support, and perception of an adequate balance between work and family life can be a rather resistant subgroup of the general Nepali population. Concurrently, the effect of masculine norms, under-reporting (possibility), and the existence of financial stresses and childhood chronic conditions suggest that low reported symptoms cannot be complacency. Regular screening and supportive measures by mental health professionals are still significant. Enhancing family/ workplace support systems, which both Nepali and international studies suggest to ensure that fathers stay well, could aid in accessing fathers who are struggling and yet would not be able to openly declare their distress or seek assistance. Despite careful planning and implementation, there are limitations associated with this study. Its design was a cross-sectional design, which means that it is unable to establish cause-and-effect relations between psychosocial factors and mental health symptoms. The study was done in one tertiary hospital in Kathmandu, and the sample only comprised fathers visiting the outpatient department, so it may not be the most valid way to generalize the results to the fathers in other locations or other areas of Nepal. While the focus on employed fathers enabled a targeted analysis of workplace and financial stressors, it limits the generalizability of the findings to unemployed fathers or those in the informal sector, such as agriculture, who may experience different psychosocial pressures. The data were self-reported, and therefore, there is a possibility of recall error or a desire to provide socially acceptable responses, particularly on sensitive topics like masculinity and mental health. Moreover, some of the variables were quantified by simple yes/no questions, and part of the continuous variables were dichotomized into two categories, which could have minimized the insight and details of the results. Although a few of the associations were found to be statistically significant, other Adjusted Odds Ratios (AORs) had large 95% confidence intervals. These large ranges indicate some lack of accuracy in the point estimates, which can probably be explained by low numbers of events in certain subgroups. Thus, on the one hand, these findings point to a strong direction of effect, but on the other hand, one should take the magnitude of the associations with caution. It is suggested that future studies should be conducted with bigger sample sizes to redefine these estimates.

## Conclusion

Fathers of children who have been treated in a tertiary hospital in Nepal demonstrated a low prevalence of depression, anxiety, and stress, reflecting low reported levels of psychological symptoms in this sample, though they should be interpreted with caution, given the potential for under-reporting in this cultural context. Nevertheless, clearly defined risk factors were revealed with multivariable analyses. The most predictable factors of depression, anxiety, and stress were recent stressful events and a lack of family support. Financial burden was connected with increased distress, especially anxiety, but it became less significant after adjustment. Close conformity to conventional masculine standards was also independently related to anxiety. Unadjusted analyses indicated a relationship between work-life imbalance, partner relationship, and chronic child health issues, but they were reduced when confounders were taken into consideration. These results indicate that, although the overall levels of symptoms are low, there is a high-risk group that is threatened by mental health, which is provoked by new stressors, poor family support, financial pressures, and strict gender-role stereotypes. Mental-health referrals in children and father-based interventions, which strengthen the family support, flexibility at work, and attitudes to seeking help, are warranted.

## Supporting information

S1 File STROBE statement. (DOCX)

S1 Data SPSS Data File. (SAV)

S2 File Inclusivity in global research. (DOCX)
